# Colonization of Supplemented *Bifidobacterium breve* M-16V in Low Birth Weight Infants and Its Effects on Their Gut Microbiota Weeks Post-administration

**DOI:** 10.3389/fmicb.2021.610080

**Published:** 2021-04-07

**Authors:** Ayako Horigome, Ken Hisata, Toshitaka Odamaki, Noriyuki Iwabuchi, Jin-zhong Xiao, Toshiaki Shimizu

**Affiliations:** ^1^Next Generation Science Institute, Morinaga Milk Industry Co., Ltd., Kanagawa, Japan; ^2^Department of Pediatrics, Juntendo University Faculty of Medicine, Tokyo, Japan; ^3^Food Ingredients and Technology Institute, Morinaga Milk Industry Co., Ltd., Kanagawa, Japan

**Keywords:** low birth weight infants, gut microbiota, *Bifidobacterium breve* M-16V, probiotics, colonization

## Abstract

The colonization and persistence of probiotics introduced into the adult human gut appears to be limited. It is uncertain, however, whether probiotics can successfully colonize the intestinal tracts of full-term and premature infants. In this study, we investigated the colonization and the effect of oral supplementation with *Bifidobacterium breve* M-16V on the gut microbiota of low birth weight (LBW) infants. A total of 22 LBW infants (12 infants in the M-16V group and 10 infants in the control group) were enrolled. *B. breve* M-16V was administrated to LBW infants in the M-16V group from birth until hospital discharge. Fecal samples were collected from each subject at weeks (3.7–9.3 weeks in the M-16V group and 2.1–6.1 weeks in the control group) after discharge. qPCR analysis showed that the administrated strain was detected in 83.3% of fecal samples in the M-16V group (at log_10_ 8.33 ± 0.99 cell numbers per gram of wet feces), suggesting that this strain colonized most of the infants beyond several weeks post-administration. Fecal microbiota analysis by 16S rRNA gene sequencing showed that the abundance of Actinobacteria was significantly higher (*P* < 0.01), whereas that of Proteobacteria was significantly lower (*P* < 0.001) in the M-16V group as compared with the control group. Notably, the levels of the administrated strain and indigenous *Bifidobacterium* bacteria were both significantly higher in the M-16V group than in the control group. Our findings suggest that oral administration of *B. breve* M-16V led to engraftment for at least several weeks post-administration and we observed a potential overall improvement in microbiota formation in the LBW infants’ guts.

## Introduction

The gut microbiota in infancy plays many important roles underpinning healthy development and thereby impacts future health. Recent studies have indicated that there is a link between infant gut dysbiosis and an increased risk of developing acute and long-term inflammatory diseases in later life such as asthma (Arrieta et al., 2015, 2018; Zimmermann et al., 2019), type 1 diabetes (Kostic et al., 2015) and obesity (Cox et al., 2014). Low birth weight (LBW) preterm infants have important differences in the composition of their intestinal microbiota when compared with full-term infants (Henderickx et al., 2019). These differences are related to immaturely-developed guts and receiving antibiotic treatments, as well as the neonatal intensive care hospital environment itself, which limits a preterm infant’s contact with commensal bacteria (Henderickx et al., 2019). The typical gut microbiota of preterm infants is characterized by the presence of potentially pathogenic bacteria commonly found in the hospital environment, such as *Klebsiella, Escherichia*, *Staphylococcus*, and *Enterococcus* (Patel et al., 2016; Stewart et al., 2016). Another characteristic is the low abundance of *Bifidobacterium* (Dalby and Hall, 2020), which is the most common genus in the normal infant gut and is thought to play pivotal roles in maintaining infant health (Leahy et al., 2005; Di Gioia et al., 2014). Combined with the underdeveloped gut and immune system in premature infants, gut dysbiosis increases their susceptibility to conditions such as sepsis and necrotizing enterocolitis (NEC) (Neu and Pammi, 2017), the latter of which is the most common and lethal gastrointestinal emergency for them. Therefore, early intervention to improve gut dysbiosis is essential for infants, especially premature ones.

Administration of probiotic *Bifidobacterium* strains is one potential approach for establishing normal gut microbiota in premature infants. Previous research studies have shown that supplementation with *Bifidobacterium* strains results in higher numbers of *Bifidobacterium* and the lower counts of *Enterobacteriaceae* in premature infants (Mohan et al., 2006; Ishizeki et al., 2013). *Bifidobacterium breve* M-16V is a probiotic strain originating from a healthy infant and has been incorporated into several products including infant formula (Wong et al., 2019). It has received GRAS status for foods including infant formula from the US Food and Drug Administration (GRAS No. 453–455). This strain has been shown to have gut microbiota modulating potential in infants and can protect against preterm- and infant-related diseases (Wong et al., 2019), although adequately powered, preferably cluster randomized controlled trials are needed to confirm these findings (Athalye-Jape et al., 2018). For example, a randomized, double-blind, placebo-controlled trial showed that *B. breve* M-16V supplementation for three weeks resulted in a significantly higher abundance of *B. breve* in the feces of preterm infants, unlike the placebo control group that had *B. breve* counts below the detection level (Patole et al., 2014). It was also reported that daily supplementation with *B. breve* M-16V decreased the incidence of NEC in very low birth weight neonates with a birth gestational age less than 34 weeks (Patole et al., 2016). The study was a retrospective cohort study involving 835 preterm neonates as historical controls and 920 preterm neonates receiving this strain. An experimental rat NEC model supported the preventive effect of *B. breve* M-16V administration on NEC and revealed that the mechanism involved modulation of Toll-like receptor expression and inflammatory response suppression (Satoh et al., 2016). To date, *B. breve* M-16V has been used to reduce the risk of preterm birth complications with LBW infants in more than 120 neonatal intensive care units (NICUs) in affiliated hospitals in Japan, Australia, New Zealand and Singapore (Umezaki et al., 2010; Patole et al., 2014, 2016; Athalye-Jape et al., 2018). As mentioned above, although live cells of *B. breve* M-16V have been confirmed to have effects on gut microbiota and infant health, little information exists about colonization of this strain in the gut and whether colonization following probiotic supplementation has a prolonged effect on gut microbiota.

Therefore, in the present study, we administered *B. breve* M-16V to LBW infants (gestation ≤ 37 weeks) admitted to NICU from birth to hospital discharge and followed up these patients for several weeks after discharge. We observed that *B. breve* M-16V colonized in the gut and contributed to a potential improvement of the gut microbiota composition in the LBW infants at least for several weeks post-administration.

## Materials and Methods

### Subjects and Sample Collection

**FIGURE 1:**
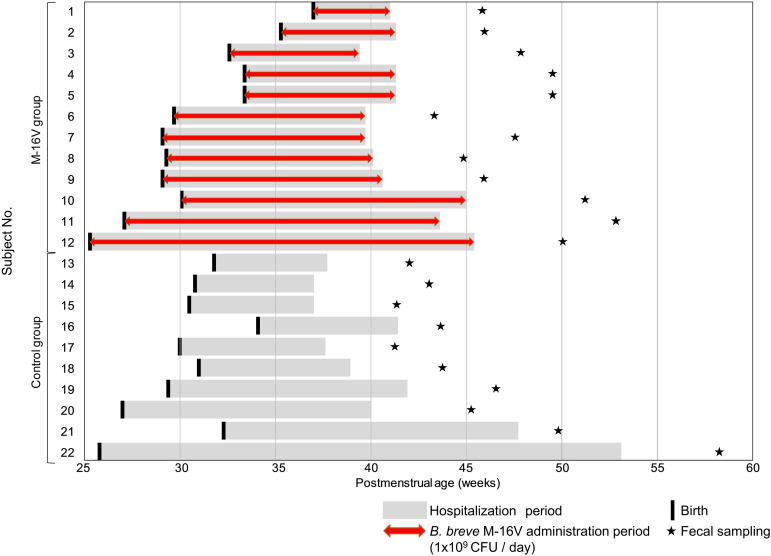
Outline of study schedule for each subject across postmenstrual age.

This study was reviewed and approved by the Ethics Committee of Juntendo University Hospital, Japan, and written informed parental consent was obtained. The outline of study schedule for each subject is shown in Figure 1. A total of 22 infants with gestational ages ≤37 weeks and birth weights <2,000 gram who were admitted to the NICU of the Juntendo University Urayasu Hospital (control group, *n* = 10) or the Juntendo University Hospital (M-16V group, *n* = 12) from March 2012 to February 2017 were enrolled. After birth, infants in the M-16V group were daily administrated a freeze-dried preparation of *B. breve* M-16V (dose, 1 × 10^9^ CFU dissolved in 4 mL of sterile water) just before feeding. The probiotic administration was continued until hospital discharge. Infants in the control group were not administrated any probiotic supplement. At 2–9 weeks after hospital discharge, fresh fecal samples were collected from each subject’s diaper to a tube. The fecal samples collected were stored below −18°C until delivery to the laboratory. Immediately upon receipt, the fecal samples were stored at −80°C until further analysis. Details of the study schedule for each subject is shown in Supplementary Table 1.

### Fecal DNA Preparation and Microbiota Analysis

DNA was extracted from the fecal samples as previously described (Sugahara et al., 2015). Purified DNA was suspended in 2,000 μl of Tris-EDTA buffer (pH 8.0).

PCR amplification and DNA sequencing of the V3–V4 region of the bacterial 16S rRNA gene was performed on the Illumina MiSeq instrument (Illumina, San Diego, CA, United States) as previously described (Odamaki et al., 2016). After removing the sequences consistent with the data from the Genome Reference Consortium human build 38 (GRCh38) and the phiX reads from the raw Illumina paired-end reads, the sequences were analyzed using the QIIME2 software package (version 2017.10)^1^. Potential chimeric sequences were removed using DADA2 (Callahan et al., 2016), and 30 and 90 bases were trimmed from the 3′ region of the forward and the reverse reads, respectively. Taxonomical classification was performed using the Naive Bayes classifier trained on the Greengenes13.8 dataset with a 99% sequence similarity threshold for full-length Operational Taxonomic Units.

### Quantitative PCR (qPCR)

The fecal DNAs as described above were applied for qPCR which was performed on the ABI PRISM 7500 Fast Real-Time PCR system (Thermo Fisher Scientific K.K., Uppsala, Sweden) with SYBR Premix Ex Taq (TaKaRa Bio, Shiga, Japan) to quantitate the *Bifidobacterium* species and strains. The primer sets used are shown in Supplementary Table 2. The primers specific for *Bifidobacterium longum*, *Bifidobacterium catenulatum*, and *Bifidobacterium adolescentis* groups have been described previously (Matsuki et al., 1998, 2004). The *B. breve* M-16V-specific primer set was designed using Primer 3 software (v.0.4.0) (Untergasser et al., 2012) after identifying unique regions in this strain by multiple sequences alignments of the complete genome sequences of *B. breve* M-16V and publically available genomes of *B. breve* strains. We confirmed the specificity of this specific primer set using other 37 *B. breve* strains in Morinaga Culture Collection.

PCR amplification was performed using the program previously described (Kato et al., 2017) with the exception of *B. breve* M-16V, whose detection consisted of an amplification program of one cycle at 95°C for 20 s, 40 cycles at 95°C for 3 s and 60°C for 30 s, and one final cycle at 95°C for 15 s. The following *Bifidobacterium* strains were used as the standards for species/strain-specific quantification: *B. breve* JCM1192^*T*^, *B. longum* subsp. *longum* JCM1217^*T*^, *Bifidobacterium bifidum* JCM1255^*T*^, *Bifidobacterium pseudocatenulatum* JCM1200^*T*^, *B. adolescentis* JCM1275^*T*^, and *B. breve* M-16V.

### Statistical Analysis

Statistical analyses were performed using EZR software ver. 1.50 (Kanda, 2013) or R software ver. 3.6.0. Intergroup differences were analyzed using the unpaired Student’s *t*-test or Welch’s *t*-test, and the Mann-Whitney *U-*test, for parametric and non-parametric data, respectively. Fisher’s exact test or χ^2^ test was conducted for categorical data. The cell number calculations for *Bifidobacterium* species or strains were substituted by log_10_ 6 per gram of wet feces for samples that fell below the detection limits. Differences in the gut microbiota profiles between the control and M-16V groups were analyzed by principal coordinate analysis (PCoA). A permutational multivariate analysis of variance (PERMANOVA) test for UniFrac distances was used for multivariate analysis to test the variation in microbiota composition explained by each factor. Associations between relative abundance of Actinobacteria or Proteobacteria and subject’s characteristics were assessed by Spearman’s rank correlation test. For all statements, *P* < 0.05 were considered to be statistically significant.

### Data Availability

DNA sequences corresponding to the 16S rRNA gene data have been deposited in the DNA Data Bank of Japan (DDBJ) under accession number DRA010463.

## Results

### General Characteristics of the Subjects

Altogether, 22 infants (10 in the control group and 12 in the M-16V group) were enrolled in this study. Table 1 shows the characteristics of the subjects. There was no significant difference in the maternal features between the groups. As neonatal features, infants were matched for gender, gestational age and breast-feeding rate, and the hospitalization period/supplementation duration, postnatal age, and corrected age at the fecal sampling were comparable between the two groups. However, birth weight and discharge weight were significantly higher in the M-16V group than in the control group. The period from hospital discharge to fecal sampling was significantly longer in the M-16V group than in the control group.

**TABLE 1:** Characteristics of the subjects.

	M-16V group (*n* = 12)	Control group (*n* = 10)	*P*-value
**Maternal features**			
Age (years)	34.0 ± 4.0	31.3 ± 3.3	0.100
C-section (*n*, %)	8 (66.7)	9 (90)	0.323
Antibiotics during labor (*n*, %)	10 (83.3)	9 (90)	1.000
GBS test (*n*, %)			
Positive	1 (8.3)	1 (10)	1.000
Negative	8 (66.7)	7 (70)	
Not done	3 (25)	2 (20)	
**Neonatal features**			
Gestational age (weeks)	31.0 ± 3.4	30.3 ± 2.4	0.613
Birth weight (g)	1350.5 ± 250.1	1096.3 ± 254.7	0.029*
Discharged weight (g)	3430.3 ± 926.1	2630.0 ± 451.1	0.017*
Male (*n*, %)	7 (58.3)	3 (30)	0.369
Chronic lung disease	1 (8.3)	1 (10)	1.000
Antibiotics exposure (*n*, %)	5 (41.7)	3 (30)	0.675
Treatment for PDA			
Clipping (*n*, %)	1 (8.3)	1 (10)	0.814
Indomethacin (*n*, %)	3 (25)	4 (40)	
Supplementation duration/hospitalization period (weeks)	10.6 ± 4.6	10.9 ± 6.6	0.887
Breast-feeding (%)^†^	54.2 ± 39.6	45.0 ± 23.0	0.526
Fecal sampling (weeks after discharge)	6.4 ± 1.9	4.3 ± 1.3	0.009**
Postnatal age at the fecal sampling (weeks)	17.0 ± 5.0	15.3 ± 6.8	0.509
Corrected age at the fecal sampling (weeks)	7.9 ± 2.8	5.5 ± 5.2	0.184

Measured variable data are expressed as the mean ± SD. Intergroup differences were analyzed using the χ^2^-test or Fisher’s exact test for categorical data and the unpaired Student’s t-test or Welch’s t-test for measured variables.*P < 0.05; **P < 0.01.

*^†^Percentage of breast milk among the total nutrition (breast milk + infant formula), as surveyed by questionnaire. GBS, Group B Streptococcus; PDA, patent ductus arteriosus.*

### Effect of *B. breve* M-16V Administration on the Gut Microbiota of the Infants

**FIGURE 2:**
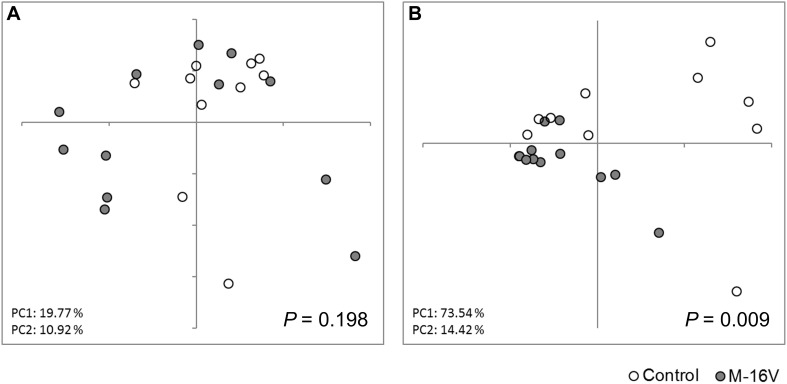
Gut microbiota profiles by PCoA. **(A)** Unweighted and **(B)** weighted UniFrac PCoA of the fecal microbiota obtained from subjects in the control group (*n* = 10) and the M-16V group (*n* = 12). Intergroup differences were analyzed using PERMANOVA.

**FIGURE 3:**
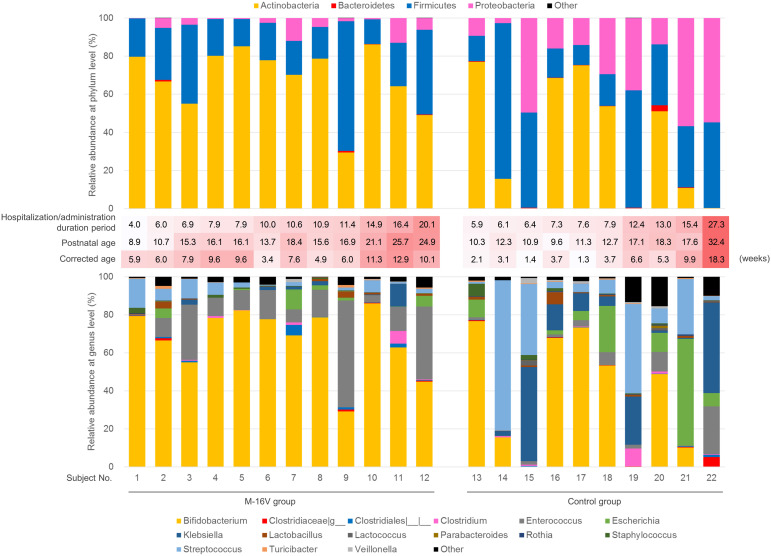
Fecal microbiota composition of each subject. Relative abundance of each phylum (upper) and genus (lower) of microbiota of each subject. The four phyla and 15 genera indicated in Table 2 were shown. In the middle of the figures, hospitalization period (that is, probiotic administration duration in the M-16V group), postnatal and corrected age at the fecal sampling scaled by red color were indicated. The darker the red color, the higher the value of administration duration/hospitalization period, postnatal age, and corrected age, respectively. Subject No. 1–12 were in the M-16V group, and subject No. 13–22 were in the control group.

To evaluate the effect of administering probiotics during early life on the composition of fecal microbiota, we collected fecal samples 3–9 weeks after their administration. No significant difference in the alpha diversity of the microbiota was observed between the groups (Supplementary Table 3). PCoA of the fecal microbiota based on the weighted UniFrac distance indicated that *B. breve* M-16V administration had an impact on the composition of the fecal microbiota (Figure 2). PERMANOVA testing revealed a significant difference in the gut microbiota profiles of the M-16V and control groups. As shown in Table 2 and Figure 3, the dominant phylum identified in the M-16V group was Actinobacteria (74%), followed by Firmicutes (19.9%). In contrast, the gut microbiota from the control group showed a higher abundance of Proteobacteria (22.7%) than that of the M-16V group (3%). The relative abundance of Actinobacteria was significantly higher, whereas that of Proteobacteria was significantly lower in the M-16V group than in the control group. At the genus level, the relative abundances of *Bifidobacterium* and *Enterococcus* were significantly higher, whereas those of *Rothia*, *Lactococcus*, and *Klebsiella* were significantly lower in the M-16V group than in the control group.

**TABLE 2:** Fecal microbiota composition.

	Median (IQRs) %		*P*-value
	
	M-16V group (*n* = 12)	Control group (*n* = 10)	
**Phylum**			
Actinobacteria	74.0 (61.9–79.8)	33.3 (3.0–64.9)	0.009**
Bacteroidetes	0.0 (0.0–0.3)	0.2 (0.1–0.3)	0.132
Firmicutes	19.9 (17.5–30.7)	31.9 (15.7–48.6)	0.628
Proteobacteria	3.0 (0.6–5.4)	22.7 (14–46.7)	0.000**
**Genus**			
*Bifidobacterium*	73.44 (60.96–78.87)	32.19 (2.56–64.28)	0.011*
*Clostridiaceae |g_*	0.08 (0.00–0.37)	0.30 (0.16–0.34)	0.366
*Clostridiales |__|__*	0.29 (0.04–0.89)	0.15 (0.05–0.25)	0.531
*Clostridium*	0.13 (0.00–0.64)	0.29 (0.00–0.75)	0.707
*Enterococcus*	11.60 (8.91–18.74)	1.90 (1.26–5.90)	0.011*
*Escherichia*	0.44 (0.00–2.99)	5.97 (0.63–9.91)	0.115
*Klebsiella*	0.22 (0.00–1.99)	7.22 (1.59–22.38)	0.015*
*Lactobacillus*	0.29 (0.08–1.03)	0.68 (0.35–0.91)	0.373
*Lactococcus*	0.00 (0.00–0.16)	0.19 (0.08–0.56)	0.044*
*Parabacteroides*	0.00 (0.00–0.13)	0.12 (0.07–0.20)	0.120
*Rothia*	0.02 (0.00–0.07)	0.24 (0.11–0.28)	0.017*
*Staphylococcus*	0.31 (0.08–0.59)	0.40 (0.04–1.36)	0.765
*Streptococcus*	2.57 (1.34–6.41)	7.61 (3.60–35.26)	0.050
*Turicibacter*	0.18 (0.05–0.43)	0.30 (0.15–0.40)	0.597
*Veillonella*	0.03 (0.00–0.18)	0.36 (0.00–0.84)	0.283

Data are expressed as the medians (IQRs) of the taxa with a median relative abundance of >0.1% in at least one group. Intergroup differences were analyzed using the Mann-Whitney U-test. *P < 0.05; **P < 0.01. IQRs, interquartile range.

We investigated the influence of factors such as gestational age and birth/discharge weight on the intestinal microbiota. There were significant correlations between the relative abundance of Actinobacteria/Proteobacteria, which were the major differences between the two groups, and some factors including birth weight and others (Supplementary Figures 1, 2). In addition, we found that factors such as gestational age and birth/discharge weight contributed a small proportion of variance, and oral administration of *B. breve* M-16V was the only significant variable explaining variance in the infant microbiota composition, by the PERMANOVA multivariate analysis using a weighted UniFrac matrix (Table 3).

**TABLE 3:** Multivariate analysis using PERMANOVA to test the variation in microbiota composition explained by each factor.

Variable	*R*^2^	*P*-value
Treatment (M-16V or Control)	0.238	0.009**
Gestational age (<30 or ≥ 30 weeks)	0.035	0.459
Birth weight (<1,200 or ≥1,200 g)	0.076	0.183
Discharge weight (<3,000 or ≥3,000 g)	0.036	0.444
Hospitalization period (<10 or ≥10 weeks)	0.029	0.527
Postnatal age at the fecal sampling (<16 or ≥ 16 weeks)	0.026	0.565
Corrected age at the fecal sampling (<7 or ≥7 weeks)	0.016	0.746
Fecal sampling (<5 or ≥5 weeks after discharge)	0.019	0.692
Residuals	0.524	
Total	1.000	

The PERMANOVA multivariate analysis was performed using a weighted UniFrac matrix.

**P < 0.01.

### Quantitative PCR Detection of Bifidobacterium Species

**FIGURE 4:**
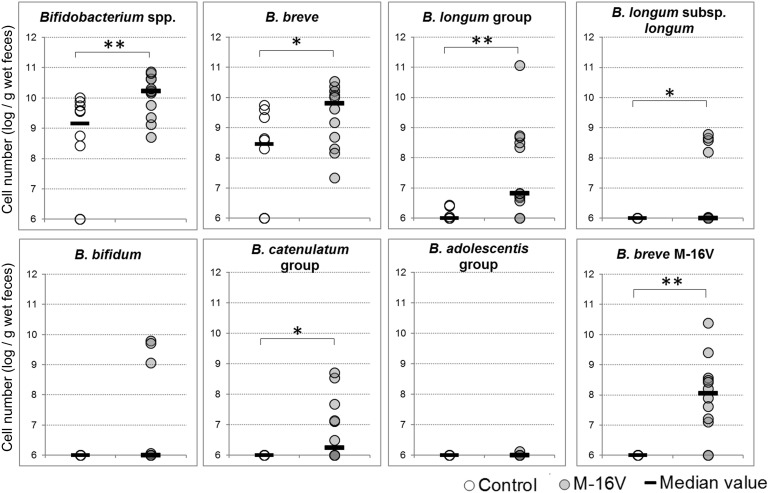
Quantitative PCR detection of *Bifidobacterium* species and *B. breve* M-16V. Cell numbers were determined as the log_10_ of cells per gram wet weight in each fecal sample. The detection limit was below 10^6^/g wet weight of feces. Intergroup differences were analyzed using the Mann-Whitney *U*-test. ^∗^*P* < 0.05; ^∗∗^*P* < 0.01.

Because the abundance difference in *Bifidobacterium* between the groups was the most remarkable, we investigated the bifidobacteria composition at the species level in addition to analyzing the *B. breve* M-16V abundance by qPCR. Notably, *B. breve* M-16V was detected in the fecal samples from all subjects in the M-16V group except for two infants (Subject Nos. 9 and 10 in Figure 3), suggesting that this strain colonized a subset of infants for at least several weeks after discontinuing the probiotics. The cell numbers for *Bifidobacterium* spp., *B. breve*, the *B. longum* group, and the *B. catenulatum* group were significantly higher in the M-16V group than in the control group (Figure 4).

## Discussion

Probiotics supplementation is a promising approach to improve dysbiosis and prevent gut microbiota-associated diseases in LBW infants. However, it is unclear whether the microbial components in probiotic treatments can persist in the gut during early life, although some studies indicated the potential for some probiotics (Frese et al., 2017; Alcon-Giner et al., 2020; Yousuf et al., 2020) as described below. This study found the colonization of *B. breve* M-16V in the intestinal tract of most LBW infants for at least several weeks following cessation of its administration as a probiotic. It has been reported that the persistence of introduced probiotics in the adult gut is limited. Most probiotic strains were only detectable for less than two weeks after the administration period despite their high detection rates in the gastrointestinal tract during probiotic treatment (Alander et al., 2001; Frese et al., 2012; Charbonneau et al., 2013). One notable study suggested a possible probiotic colonization in 30% of the adult subjects for up to 6 months after administration (Maldonado-Gómez et al., 2016). In contrast with adults, whose gut microbiota remains relatively stable (Faith et al., 2013), the composition of the gut microbiota in infants reportedly shows great shifts up to 2–4 years old when it reaches a more stable and mature composition (Voreades et al., 2014; Odamaki et al., 2016; Stewart et al., 2018). Furthermore, the microbiota composition is more immature and less stable in premature infants than full-term infants (Gritz and Bhandari, 2015; Henderickx et al., 2019). This instability of the gut microbiota in addition to the low abundance of *Bifidobacterium* potentially provided a niche opportunity for oral administration of bifidobacteria probiotic strains such as *B. breve* M-16V in LBW infants.

Another reason for the high colonization of *B. breve* M-16V might arise from the species-specific property. Bifidobacteria display a difference in their ecological adaptation among species and show genotypic and physiological differences related to their different residential origin. Bifidobacterial species of human origin are grouped as human-residential bifidobacteria (HRB) (Odamaki et al., 2015; Wong et al., 2018). Among HRB, *B. breve*, *B. longum* subsp. *infantis*, *B. longum* subsp. *longum*, and *B. bifidum*, which are prevalently isolated from the infant’s intestine, are referred to as infant-type HRB. Unlike non-HRB such as *Bifidobacterium animalis* subsp. *lactis* and *Bifidobacterium thermophilum*, infant-type HRB have been reported to possess high ability to assimilate human milk oligosaccharides (HMOs) and undergo specific adaptation to the infant host (Wong et al., 2018). Indeed, Underwood et al. (2013) showed that *B. longum* subsp. *infantis* colonization was better than *B. animalis* subsp. *lactis* colonization in both formula-fed and human milk-fed premature infants. In human milk-fed infants, greater increases in fecal *Bifidobacterium* and decreases in γ-Proteobacteria followed the administration of *B. longum* subsp. *infantis* than that of *B. animalis* subsp. *lactis* (Underwood et al., 2013). Another study confirmed that *B. breve* or *B. longum* subsp. *infantis* were early colonizers apparently independent of early life-events, such as mode of delivery and type of feeding, while the colonization of *B. animalis* subsp. *lactis* was dependent solely on the type of feeding (Martin et al., 2016). The authors suggested that the frequent colonization by *B. animalis* subsp. *lactis* in infants exposed to formula feeding may result from the use of formula supplemented with probiotic strains belonging to this subspecies. These findings suggest that infant-type HRB are more effective colonizers of the infant gut. Consistent with this finding, it was shown the persistent colonization of probiotic strains of *B. longum*, *B. bifidum*, and *B. breve* up to 5 months after supplementation of commercially probiotics containing these *Bifidobacterium* strains and *Lactobacillus rhamnosus* strain in preterm infants (Yousuf et al., 2020). Another study also indicated that the administration of *B. longum* subsp. *infants* resulted in a colonization period of at least a month in the breast-fed infant gut (Frese et al., 2017). The authors postulated that the colonization of this strain was attributed to the ancient adaptations of *B. longum* subsp. *infantis* to HMOs; that is, the capacity to transport these substances into this bacterium’s cytoplasm and consume the full range of HMOs (Underwood et al., 2015). Through their study on probiotic supplementation of preterm infants (Alcon-Giner et al., 2020), suggested the ability of the probiotic *B. bifidum* strain to colonize the preterm infants’ gut by showing its presence in two fecal samples collected at 41 and 50 days after supplementation. Noteworthy, genomic analysis of this strain showed the presence of genes involved in HMO utilization and mucin degradation which may aid the gut persistence (Alcon-Giner et al., 2020). In the present study, *B. breve* M-16V was detected in 83.3% of fecal samples from LBW infants in M-16V group at 3.7–9.3 weeks following its cessation of administration. Unfortunately, there were too few subjects to evaluate the influence of the infant feeding methods on the colonization of *B. breve* M-16V in this study; however, we noted that the two samples from the infants where *B. breve* M-16V was not detected were mixed-fed with breast milk and infant formula (data not shown). Further studies are needed to investigate the environmental factors that could affect the colonization of probiotics (e.g., whether or not the maternal genetic background related to HMO secretion or the feeding method affects colonization).

Recent studies have shown that the introduction of live microbes does not result in significant alterations of the fecal microbiota in healthy adults (Kim et al., 2013; Kristensen et al., 2016). Studies on infants are controversial with respect to this finding. Ishizeki et al. (2013) reported that the supplementation of single (*B. breve* M-16V) or multiple (*B. breve* M-16V, *B. longum* subsp. *infantis* M-63, and *B. longum* subsp. *longum* BB536) infant-type HRB strains to LBW infants resulted in the increase of the detection rate and number of *Bifidobacterium* in feces. Also, a study by Plummer et al. (2018) indicated that probiotic supplementation with *B. longum* subsp. *infantis* BB-02, *Streptococcus thermophilus* TH-4 and *B. animalis* subsp. *lactis* BB-12 from soon after birth increased the abundance of *Bifidobacterium* in the gut microbiota of very preterm infants during supplementation period. Furthermore, potential long-term contribution of probiotic strains on development of gut microbiota in preterm infants have been indicated by some researches (Alcon-Giner et al., 2020; Yousuf et al., 2020). On the other hand, a double-blind, randomized placebo-controlled intervention showed that intake of *Lactobacillus acidophilus* NCFM or *B. animalis* subsp. *lactis* Bi-07 to young children with atopic dermatitis for eight weeks did not affect the composition and diversity of the main bacterial populations in feces (Larsen et al., 2011). Similarly, no effect on the overall microbiota composition was observed when *Lactobacillus reuteri* DSM 17938 was administrated to breast-fed colicky infants for 21 days (Roos et al., 2013). We found that *B. breve* M-16V administration significantly impacted the overall microbiota composition beyond the non-administration period of 3.7–9.3 weeks. In the M-16V group, the relative abundance of Proteobacteria was significantly lower than in the control group. A sustained increase in Proteobacteria abundance is considered a signature of dysbiosis (Shin et al., 2015). Some reports have indicated an association between intestinal Proteobacteria and NEC in premature infants (Wang et al., 2009; Pammi et al., 2017; Lindberg et al., 2020). Mirpuri et al. (2014) found that the IgA-dependent suppression of Proteobacteria in the infant gut was important for establishing a beneficial commensal population and reducing susceptibility to colonic injury and inflammation. At the genus level, the relative abundance of *Klebsiella*, which have been associated with neonatal bacterial infections (Podschun and Ullmann, 1998; Hornik et al., 2012) and NEC (Sim et al., 2015; Olm et al., 2019), were lower in the M-16V group than in the control group. Contrastingly, the relative abundance of *Bifidobacterium* was significantly higher in the M-16V group than in the control group. It has been reported that a higher abundance of *Bifidobacterium* in early infancy is associated with better immune system responses to vaccination, potentially enhancing immunological memory (Huda et al., 2019). Conversely, a lower abundance of *Bifidobacteriaceae*, which primarily includes the *Bifidobacterium* genus, is suggested to trigger the development of allergic sensitization, eczema, or asthma (Zimmermann et al., 2019).

Factors such as gestational age and birth weight have been reported to affect the developing gut microbiota in preterm neonates (Korpela et al., 2018; Henderickx et al., 2019; Alcon-Giner et al., 2020). Some factors including birth weight had significant correlations with the relative abundance of Actinobacteria/Proteobacteria. A part of these correlations might be due to the significant difference of the birth body weight between groups, and the close association among the birth weight, the gestational age, and the hospitalization period (Supplementary Figure 3). Our PERMANOVA multivariate analysis confirmed that the effect of the supplementation of *B. breve* M-16V on the gut microbiota was greater than that of other factors such as gestational age and birth weight.

Our qPCR analysis revealed that the cell numbers of the administrated strain and some of the indigenous bifidobacteria species were significantly higher in the M-16V group. It remains unclear as to why administering *B. breve* M-16V promoted the colonization of other bifidobacteria, but one possibility is that because acetic acid is the main metabolite it might suppress the growth of acid-sensitive bacteria such as Proteobacteria, thereby providing an appropriate environment for bifidobacteria growth. Overall, our findings suggest that *B. breve* M-16V administration can contribute to the establishment of a healthy gut microbiota composition in LBW infants.

There are several limitations in this study. First, this study is not a randomized controlled trial and included a small number of infants. Second, the birth weight and hospital discharge weight were significantly higher in the M-16V group than in the control group. Body weight is an important indicator of infant maturity and a key factor influencing the intestinal microbiota in neonates, that cannot be ignored especially in LBW infants. Third, the period from the discharge, that is, the cessation of probiotic administration in the M-16V group to the fecal sampling was significantly longer in the M-16V group than the control group, though the postnatal and the corrected age at sampling were not significantly different between the groups. Since the composition of the gut microbiota dramatically changes in the early life stage, the difference of the period from the hospital discharge to the fecal sampling could lead to the difference in the gut microbiota composition. However, it is assumed that the longer non-administration period in the M-16V group would not bring the overestimation of the colonization of *B. breve* M-16V in the infant’s gut. Fourth, subjects in the two groups were from different hospitals, and the inclusion period lasted over 5 years. Such a long period might lead to change the hospital environment. The establishment of gut microbiota in very preterm infants is unstable and susceptible to the environment factors (Brooks et al., 2014) and the NICU practices (Rozé et al., 2020). Hence, a large-scale, double-blind, placebo-controlled study in the matched infants in the same facility with sequential fecal sampling from birth will be necessary to confirm the beneficial effects of *B. breve* M-16V that have been suggested in this study.

In conclusion, our results show that oral administration of *B. breve* M-16V leads to its colonization in the infant gut for at least several weeks after administration and potentially contributes to improved gut microbiota establishment. Further follow-up investigations will help to elucidate the durability of these effects through later childhood, and whether these effects carry implications for overall health later in life.

## Data Availability Statement

The datasets presented in this study can be found in online repositories. The names of the repository/repositories and accession number(s) can be found below: https://www.ddbj.nig.ac.jp/, DRA010463.

## Ethics Statement

The studies involving human participants were reviewed and approved by the Ethics Committee of Juntendo University Hospital. Written informed consent to participate in this study was provided by the participants’ legal guardian/next of kin.

## Author Contributions

J-ZX and TS conceived and designed the study. AH, NI, and KH performed the clinical trial and experiments. AH and TO analyzed the data and wrote the manuscript. TS supervised the overall study. All authors have read and approved the final manuscript.

## Conflict of Interest

AH, TO, NI, and J-ZX were employees of Morinaga Milk Industry Co., Ltd. The remaining authors declare that the research was conducted in the absence of any commercial or financial relationships that could be construed as a potential conflict of interest.

## Footnotes

1 https://qiime2.org/

## Supplementary Material

Supplementary Figure 1 Relationship between Actinobacteria and subject’s characteristics. Relationships between the relative abundance of Actinobacteria and the gestational age **(A)**, the birth weight **(B)**, the discharge weight **(C)**, the hospitalization/probiotic administration period **(D)**, the postnatal age at the fecal sampling **(E)**, the corrected age at the fecal sampling **(F)**, or the fecal sampling timing **(G)** in all subjects (black dashed line), the M-16V group (red circles and line), or the control group (blue circles and line). Associations were assessed by Spearman’s rank correlation test. **P* < 0.05.

Supplementary Figure 2 Relationship between Proteobacteria and subject’s characteristics. Relationships between the relative abundance of Proteobacteria and the gestational age **(A)**, the birth weight **(B)**, the discharge weight **(C)**, the hospitalization/probiotic administration period **(D)**, the postnatal age at the fecal sampling **(E)**, the corrected age at the fecal sampling **(F)**, or the fecal sampling timing **(G)** in all subjects (black dashed line), the M-16V group (red circles and line), or the control group (blue circles and line). Associations were assessed by Spearman’s rank correlation test. **P* < 0.05; ***P* < 0.01.

Supplementary Figure 3 Relationship between the birth weight, gestational age, and hospitalization. Relationships between the birth weight and gestational age **(A)**, the birth weight and the hospitalization period **(B)**, and the gestational age and the hospitalization period **(C)** in all subjects (black dashed line), the M-16V group (red circles and line), or the control group (blue circles and line). Associations were assessed by Spearman’s rank correlation test. **P* < 0.05; ***P* < 0.01.

Supplementary Table 1 Study schedule for each subject.

Supplementary Table 2 Quantitative PCR primer sets.

Supplementary Table 3 Alpha-diversity of the gut microbiota.

## References

[B1] Alander M. Mättö J. Kneifel W. Johansson M. Kögler B. Crittenden R. (2001). Effect of galacto-oligosaccharide supplementation on human faecal microflora and on survival and persistence of *Bifidobacterium lactis* Bb-12 in the gastrointestinal tract. *Int. Dairy J.* 11 817–825. 10.1016/S0958-6946(01)00100-5

[B2] Alcon-Giner C. Dalby M. J. Caim S. Ketskemety J. Shaw A. Sim K. (2020). Microbiota supplementation with *Bifidobacterium* and *Lactobacillus* modifies the preterm infant gut microbiota and metabolome: an observational study. *Cell Rep. Med.* 1:100077. 10.1016/j.xcrm.2020.100077 32904427 PMC7453906

[B3] Arrieta M. C. Arévalo A. Stiemsma L. Dimitriu P. Chico M. E. Loor S. (2018). Associations between infant fungal and bacterial dysbiosis and childhood atopic wheeze in a nonindustrialized setting. *J. Allergy Clin. Immunol.* 142 424–434. 10.1016/j.jaci.2017.08.041 29241587 PMC6075469

[B4] Arrieta M.-C. Stiemsma L. T. Dimitriu P. A. Thorson L. Russell S. Yurist-Doutsch S. (2015). Early infancy microbial and metabolic alterations affect risk of childhood asthma. *Sci. Transl. Med.* 7:307ra152. 10.1126/scitranslmed.aab2271 26424567

[B5] Athalye-Jape G. Rao S. Simmer K. Patole S. (2018). *Bifidobacterium breve* M-16V as a probiotic for preterm infants: a strain-specific systematic review. *J. Parenter. Enter. Nutr.* 42 677–688. 10.1177/0148607117722749 28796951

[B6] Brooks B. Firek B. A. Miller C. S. Sharon I. Thomas B. C. Baker R. (2014). Microbes in the neonatal intensive care unit resemble those found in the gut of premature infants. *Microbiome* 2:1. 10.1186/2049-2618-2-1 24468033 PMC4392516

[B7] Callahan B. J. McMurdie P. J. Rosen M. J. Han A. W. Johnson A. J. A. Holmes S. P. (2016). DADA2: high-resolution sample inference from Illumina amplicon data. *Nat. Methods* 13 581–583. 10.1038/nmeth.3869 27214047 PMC4927377

[B8] Charbonneau D. Gibb R. D. Quigley E. M. M. (2013). Fecal excretion of *Bifidobacterium infantis* 35624 and changes in fecal microbiota after eight weeks of oral supplementation with encapsulated probiotic. *Gut Microbes* 4 201–211. 10.4161/gmic.24196 23549409 PMC3669165

[B9] Cox L. M. Yamanishi S. Sohn J. Alekseyenko A. V. Leung M. Cho I. (2014). Altering the intestinal microbiota during a critical developmental window has lasting metabolic consequences. *Cell* 158 705–721. 10.1016/j.cell.2014.05.052 25126780 PMC4134513

[B10] Dalby M. J. Hall L. J. (2020). Recent advances in understanding the neonatal microbiome. *F1000Research* 9:422. 10.12688/f1000research.22355.1 32518631 PMC7255898

[B11] Di Gioia D. Aloisio I. Mazzola G. Biavati B. (2014). Bifidobacteria: their impact on gut microbiota composition and their applications as probiotics in infants. *Appl. Microbiol. Biotechnol.* 98 563–577. 10.1007/s00253-013-5405-9 24287935

[B12] Faith J. J. Guruge J. L. Charbonneau M. Subramanian S. Seedorf H. Goodman A. L. (2013). The long-term stability of the human gut microbiota. *Science* 341:1237439. 10.1126/science.1237439 23828941 PMC3791589

[B13] Frese S. A. Hutkins R. Walter J. (2012). Comparison of the colonization ability of autochthonous and allochthonous strains of lactobacilli in the human gastrointestinal tract. *Adv. Microbiol.* 2 399–409. 10.4236/aim.2012.23051

[B14] Frese S. A. Hutton A. A. Contreras L. N. Shaw C. A. Palumbo M. C. Casaburi G. (2017). Persistence of supplemented *Bifidobacterium longum* subsp. infantis EVC001 in breastfed infants. *mSphere* 2 e501–e517. 10.1128/mSphere.00501-17 29242832 PMC5717325

[B15] Gritz E. C. Bhandari V. (2015). The human neonatal gut microbiome: a brief review. *Front. Pediatr.* 3:17. 10.3389/fped.2015.00017 25798435 PMC4350424

[B16] Henderickx J. G. E. Zwittink R. D. van Lingen R. A. Knol J. Belzer C. (2019). The preterm gut microbiota: an inconspicuous challenge in nutritional neonatal care. *Front. Cell. Infect. Microbiol.* 9:85. 10.3389/fcimb.2019.00085 31001489 PMC6454191

[B17] Hornik C. P. Fort P. Clark R. H. Watt K. Benjamin D. K. Smith P. B. (2012). Early and late onset sepsis in very-low-birth-weight infants from a large group of neonatal intensive care units. *Early Hum. Dev.* 88 S69–S74. 10.1016/S0378-3782(12)70019-1 22633519 PMC3513766

[B18] Huda M. N. Ahmad S. M. Alam M. J. Khanam A. Kalanetra K. M. Taft D. H. (2019). *Bifidobacterium* abundance in early infancy and vaccine response at 2 years of age. *Pediatrics* 143:e20181489. 10.1542/peds.2018-1489 30674610 PMC6361348

[B19] Ishizeki S. Sugita M. Takata M. Yaeshima T. (2013). Effect of administration of bifidobacteria on intestinal microbiota in low-birth-weight infants and transition of administered bifidobacteria: a comparison between one-species and three-species administration. *Anaerobe* 23 38–44. 10.1016/j.anaerobe.2013.08.002 23988359

[B20] Kanda Y. (2013). Investigation of the freely available easy-to-use software “EZR” for medical statistics. *Bone Marrow Transplant.* 48 452–458. 10.1038/bmt.2012.244 23208313 PMC3590441

[B21] Kato K. Odamaki T. Mitsuyama E. Sugahara H. Xiao J. Z. Osawa R. (2017). Age-related changes in the composition of gut *Bifidobacterium* species. *Curr. Microbiol.* 74 987–995. 10.1007/s00284-017-1272-4 28593350 PMC5486783

[B22] Kim S.-W. Suda W. Kim S. Oshima K. Fukuda S. Ohno H. (2013). Robustness of gut microbiota of healthy adults in response to probiotic intervention revealed by high-throughput pyrosequencing. *DNA Res.* 20 241–253. 10.1093/dnares/dst006 23571675 PMC3686430

[B23] Korpela K. Blakstad E. W. Moltu S. J. Strømmen K. Nakstad B. Rønnestad A. E. (2018). Intestinal microbiota development and gestational age in preterm neonates. *Sci. Rep.* 8:2453. 10.1038/s41598-018-20827-x 29410448 PMC5802739

[B24] Kostic A. D. Gevers D. Siljander H. Vatanen T. Peet A. Tillmann V. (2015). The dynamics of the human infant gut microbiome in development and in progression towards type 1 diabetes. *Cell Host Microbe* 17 260–273. 10.1016/j.chom.2015.01.001 25662751 PMC4689191

[B25] Kristensen N. B. Bryrup T. Allin K. H. Nielsen T. Hansen T. H. Pedersen O. (2016). Alterations in fecal microbiota composition by probiotic supplementation in healthy adults: a systematic review of randomized controlled trials. *Genome Med.* 8:52. 10.1186/s13073-016-0300-5 27159972 PMC4862129

[B26] Larsen N. Vogensen F. K. Gøbel R. Michaelsen K. F. Abu Al-Soud W. Sørensen S. J. (2011). Predominant genera of fecal microbiota in children with atopic dermatitis are not altered by intake of probiotic bacteria *Lactobacillus acidophilus* NCFM and *Bifidobacterium animalis* subsp. lactis Bi-07. *FEMS Microbiol. Ecol.* 75 482–496. 10.1111/j.1574-6941.2010.01024.x 21204871

[B27] Leahy S. C. Higgins D. G. Fitzgerald G. F. van Sinderen D. (2005). Getting better with bifidobacteria. *J. Appl. Microbiol.* 98 1303–1315. 10.1111/j.1365-2672.2005.02600.x 15916644

[B28] Lindberg T. P. Caimano M. J. Hagadorn J. I. Bennett E. M. Maas K. Brownell E. A. (2020). Preterm infant gut microbial patterns related to the development of necrotizing enterocolitis. *J. Matern. Neonatal Med.* 33 349–358. 10.1080/14767058.2018.1490719 29909714

[B29] Maldonado-Gómez M. X. Martínez I. Bottacini F. O’Callaghan A. Ventura M. van Sinderen D. (2016). Stable engraftment of *Bifidobacterium longum* AH1206 in the human gut depends on individualized features of the resident microbiome. *Cell Host Microbe* 20 515–526. 10.1016/j.chom.2016.09.001 27693307

[B30] Martin R. Makino H. Yavuz A. C. Ben-Amor K. Roelofs M. Ishikawa E. (2016). Early-life events, including mode of delivery and type of feeding, siblings and gender, shape the developing gut microbiota. *PLoS One* 11:e0158498. 10.1371/journal.pone.0158498 27362264 PMC4928817

[B31] Matsuki T. Watanabe K. Fujimoto J. Kado Y. Takada T. Matsumoto K. (2004). Quantitative PCR with 16S rRNA-gene-targeted species-specific primers for analysis of human intestinal bifidobacteria. *Appl. Environ. Microbiol.* 70 167–173. 10.1128/aem.70.1.167 14711639 PMC321263

[B32] Matsuki T. Watanabe K. Tanaka R. Oyaizu H. (1998). Rapid identification of human intestinal bifidobacteria by 16S rRNA-targeted species- and group-specific primers. *FEMS Microbiol. Lett.* 167 113–121. 10.1111/j.1574-6968.1998.tb13216.x 9809413

[B33] Mirpuri J. Raetz M. Sturge C. R. Wilhelm C. L. Benson A. Savani R. C. (2014). *Proteobacteria*-specific IgA regulates maturation of the intestinal microbiota. *Gut Microbes* 5 28–39. 10.4161/gmic.26489 24637807 PMC4049932

[B34] Mohan R. Koebnick C. Schildt J. Schmidt S. Mueller M. Possner M. (2006). Effects of *Bifidobacterium lactis* Bb12 supplementation on intestinal microbiota of preterm infants: a double-blind, placebo-controlled, randomized study. *J. Clin. Microbiol.* 44 4025–4031. 10.1128/JCM.00767-06 16971641 PMC1698302

[B35] Neu J. Pammi M. (2017). Pathogenesis of NEC: impact of an altered intestinal microbiome. *Semin. Perinatol.* 41 29–35. 10.1053/j.semperi.2016.09.015 27986328

[B36] Odamaki T. Horigome A. Sugahara H. Hashikura N. Minami J. Xiao J. (2015). Comparative genomics revealed genetic diversity and species/strain-level differences in carbohydrate metabolism of three probiotic bifidobacterial species. *Int. J. Genomics* 2015:567809. 10.1155/2015/567809 26236711 PMC4506816

[B37] Odamaki T. Kato K. Sugahara H. Hashikura N. Takahashi S. Xiao J. (2016). Age-related changes in gut microbiota composition from newborn to centenarian: a cross-sectional study. *BMC Microbiol.* 16:90. 10.1186/s12866-016-0708-5 27220822 PMC4879732

[B38] Olm M. R. Bhattacharya N. Crits-Christoph A. Firek B. A. Baker R. Song Y. S. (2019). Necrotizing enterocolitis is preceded by increased gut bacterial replication, *Klebsiella*, and fimbriae-encoding bacteria. *Sci. Adv.* 5:eaax5727. 10.1126/sciadv.aax5727 31844663 PMC6905865

[B39] Pammi M. Cope J. Tarr P. I. Warner B. B. Morrow A. L. Mai V. (2017). Intestinal dysbiosis in preterm infants preceding necrotizing enterocolitis: a systematic review and meta-analysis. *Microbiome* 5:31. 10.1186/s40168-017-0248-8 28274256 PMC5343300

[B40] Patel A. L. Mutlu E. A. Sun Y. Koenig L. Green S. Jakubowicz A. (2016). Longitudinal survey of microbiota in hospitalized preterm very-low-birth-weight infants. *J. Pediatr. Gastroenterol. Nutr.* 62 292–303. 10.1097/MPG.0000000000000913 26230901 PMC4724288

[B41] Patole S. K. Rao S. C. Keil A. D. Nathan E. A. Doherty D. A. Simmer K. N. (2016). Benefits of *Bifidobacterium breve* M-16V supplementation in preterm neonates - a retrospective cohort study. *PLoS One* 11:e0150775. 10.1371/journal.pone.0150775 26953798 PMC4783036

[B42] Patole S. Keil A. Chang A. Nathan E. Doherty D. Simmer K. (2014). Effect of *Bifidobacterium breve* M-16V supplementation on fecal bifidobacteria in preterm neonates - a randomised double blind placebo controlled trial. *PLoS One* 9:e89511. 10.1371/journal.pone.0089511 24594833 PMC3940439

[B43] Plummer E. L. Bulach D. M. Murray G. L. Jacobs S. E. Tabrizi S. N. Garland S. M. (2018). Gut microbiota of preterm infants supplemented with probiotics: sub-study of the ProPrems trial. *BMC Microbiol.* 18:184. 10.1186/s12866-018-1326-1 30424728 PMC6234596

[B44] Podschun R. Ullmann U. (1998). *Klebsiella* spp. as nosocomial pathogens: epidemiology, taxonomy, typing methods, and pathogenicity factors. *Clin. Microbiol. Rev.* 11 589–603. 9767057 10.1128/cmr.11.4.589 PMC88898

[B45] Roos S. Dicksved J. Tarasco V. Locatelli E. Ricceri F. Grandin U. (2013). 454 pyrosequencing analysis on faecal samples from a randomized DBPC trial of colicky infants treated with *Lactobacillus reuteri* DSM 17938. *PLoS One* 8:e56710. 10.1371/journal.pone.0056710 23468874 PMC3585302

[B46] Rozé J.-C. Ancel P.-Y. Marchand-Martin L. Rousseau C. Montassier E. Monot C. (2020). Assessment of neonatal intensive care unit practices and preterm newborn gut microbiota and 2-year neurodevelopmental outcomes. *JAMA Netw. Open* 3:e2018119. 10.1001/jamanetworkopen.2020.18119 32965499 PMC7512059

[B47] Satoh T. Izumi H. Iwabuchi N. Odamaki T. Namba K. Abe F. (2016). *Bifidobacterium breve* prevents necrotising enterocolitis by suppressing inflammatory responses in a preterm rat model. *Benef. Microbes* 7 75–82. 10.3920/BM2015.0035 26420070

[B48] Shin N. R. Whon T. W. Bae J. W. (2015). *Proteobacteria*: microbial signature of dysbiosis in gut microbiota. *Trends Biotechnol.* 33 496–503. 10.1016/j.tibtech.2015.06.011 26210164

[B49] Sim K. Shaw A. G. Randell P. Cox M. J. McClure Z. E. Li M.-S. (2015). Dysbiosis anticipating necrotizing enterocolitis in very premature infants. *Clin. Infect. Dis.* 60 389–397. 10.1093/cid/ciu822 25344536 PMC4415053

[B50] Stewart C. J. Ajami N. J. O’Brien J. L. Hutchinson D. S. Smith D. P. Wong M. C. (2018). Temporal development of the gut microbiome in early childhood from the TEDDY study. *Nature* 562 583–588. 10.1038/s41586-018-0617-x 30356187 PMC6415775

[B51] Stewart C. J. Embleton N. D. Marrs E. C. L. Smith D. P. Nelson A. Abdulkadir B. (2016). Temporal bacterial and metabolic development of the preterm gut reveals specific signatures in health and disease. *Microbiome* 4:67. 10.1186/s40168-016-0216-8 28034304 PMC5200962

[B52] Sugahara H. Odamaki T. Hashikura N. Abe F. Xiao J. (2015). Differences in folate production by bifidobacteria of different origins. *Biosci. Microbiota Food Health* 34 87–93. 10.12938/bmfh.2015-003 26594608 PMC4654071

[B53] Umezaki H. Shinohara K. Satoh Y. Shoji H. Satoh H. Ohtsuka Y. (2010). Bifidobacteria prevents preterm infants from developing infection and sepsis. *Int. J. Probiotics Prebiotics* 5 33–36.

[B54] Underwood M. A. German J. B. Lebrilla C. B. Mills D. A. (2015). *Bifidobacterium longum* subspecies infantis: champion colonizer of the infant gut. *Pediatr. Res.* 77 229–235. 10.1038/pr.2014.156 25303277 PMC4350908

[B55] Underwood M. A. Kalanetra K. M. Bokulich N. A. Lewis Z. T. Mirmiran M. Tancredi D. J. (2013). A comparison of two probiotic strains of bifidobacteria in premature infants. *J. Pediatr.* 163 1585–1591.e9. 10.1016/j.jpeds.2013.07.017 23993139 PMC3842430

[B56] Untergasser A. Cutcutache I. Koressaar T. Ye J. Faircloth B. C. Remm M. (2012). Primer3–new capabilities and interfaces. *Nucleic Acids Res.* 40:e115. 10.1093/nar/gks596 22730293 PMC3424584

[B57] Voreades N. Kozil A. Weir T. L. (2014). Diet and the development of the human intestinal microbiome. *Front. Microbiol.* 5:494. 10.3389/fmicb.2014.00494 25295033 PMC4170138

[B58] Wang Y. Hoenig J. D. Malin K. J. Qamar S. Petrof E. O. Sun J. (2009). 16S rRNA gene-based analysis of fecal microbiota from preterm infants with and without necrotizing enterocolitis. *ISME J.* 3 944–954. 10.1038/ismej.2009.37 19369970 PMC2713796

[B59] Wong C. B. Iwabuchi N. Xiao J. Z. (2019). Exploring the science behind *Bifidobacterium breve* M-16V in infant health. *Nutrients* 11:1724. 10.3390/nu11081724 31349739 PMC6723912

[B60] Wong C. B. Sugahara H. Odamaki T. Xiao J. Z. (2018). Different physiological properties of human-residential and non-human-residential bifidobacteria in human health. *Benef. Microbes* 9 111–122. 10.3920/BM2017.0031 28969444

[B61] Yousuf E. I. Carvalho M. Dizzell S. E. Kim S. Gunn E. Twiss J. (2020). Persistence of suspected probiotic organisms in preterm infant gut microbiota weeks after probiotic supplementation in the NICU. *Front. Microbiol.* 11:574137. 10.3389/fmicb.2020.574137 33117319 PMC7552907

[B62] Zimmermann P. Messina N. Mohn W. W. Finlay B. B. Curtis N. (2019). Association between the intestinal microbiota and allergic sensitization, eczema, and asthma: a systematic review. *J. Allergy Clin. Immunol.* 143 467–485. 10.1016/j.jaci.2018.09.025 30600099

