# Supplementary figures and images for: Colonization of Supplemented Bifidobacterium breve M-16V in Low Birth Weight Infants and Its Effects on Their Gut Microbiota Weeks Post-administration
Source: Front Microbiol. 2021 Apr 7;12:610080. doi: 10.3389/fmicb.2021.610080 (PMC8058467; doi:10.3389/fmicb.2021.610080)

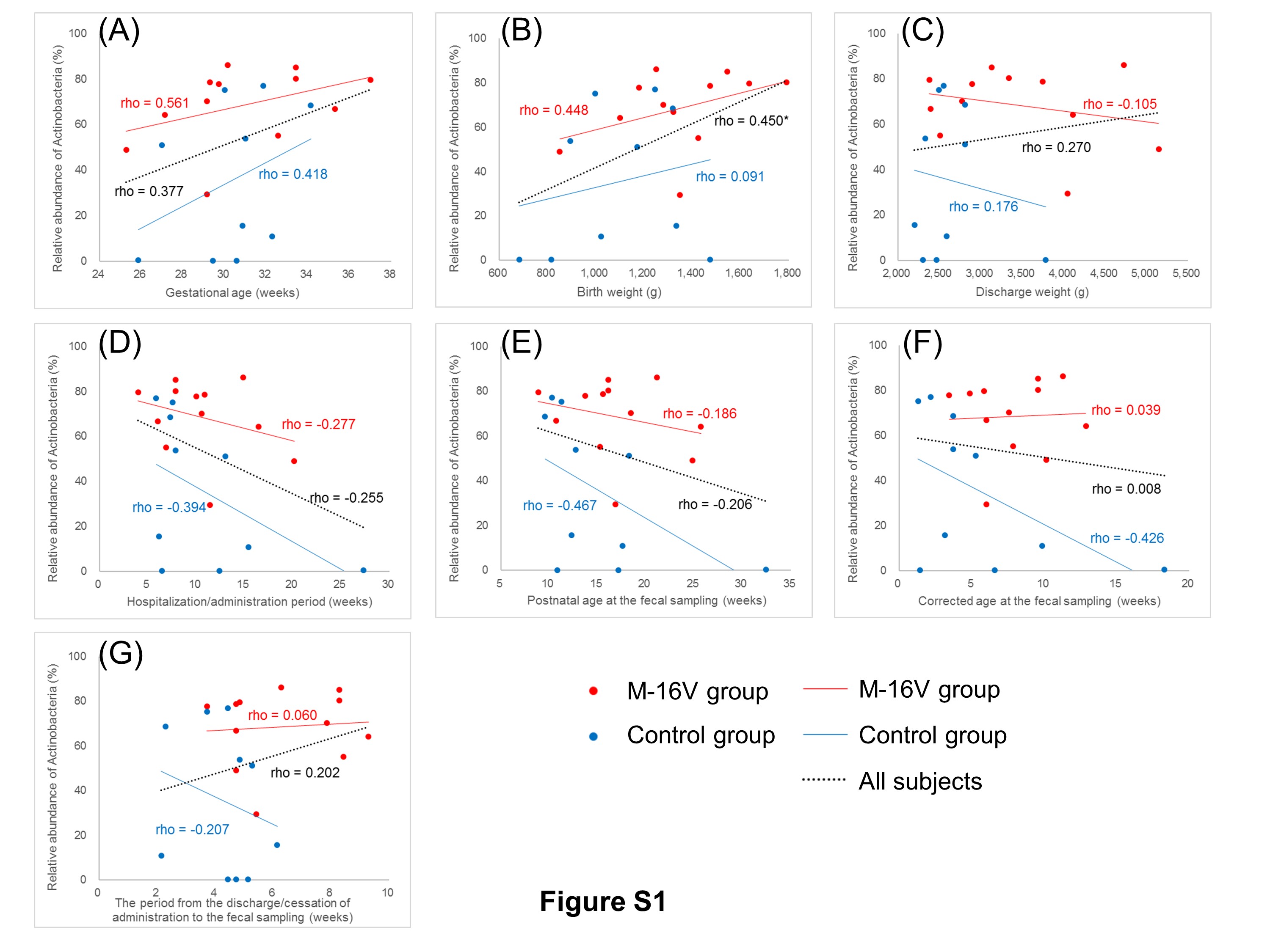

Supplement: Supplementary Figure 1 — Relationship between Actinobacteria and subject’s characteristics. Relationships between the relative abundance of Actinobacteria and the gestational age (A), the birth weight (B), the discharge weight (C), the hospitalization/probiotic administration period (D), the postnatal age at the fecal sampling (E), the corrected age at the fecal sampling (F), or the fecal sampling timing (G) in all subjects (black dashed line), the M-16V group (red circles and line), or the control group (blue circles and line). Associations were assessed by Spearman’s rank correlation test. *P < 0.05. [file Image_1.JPEG]

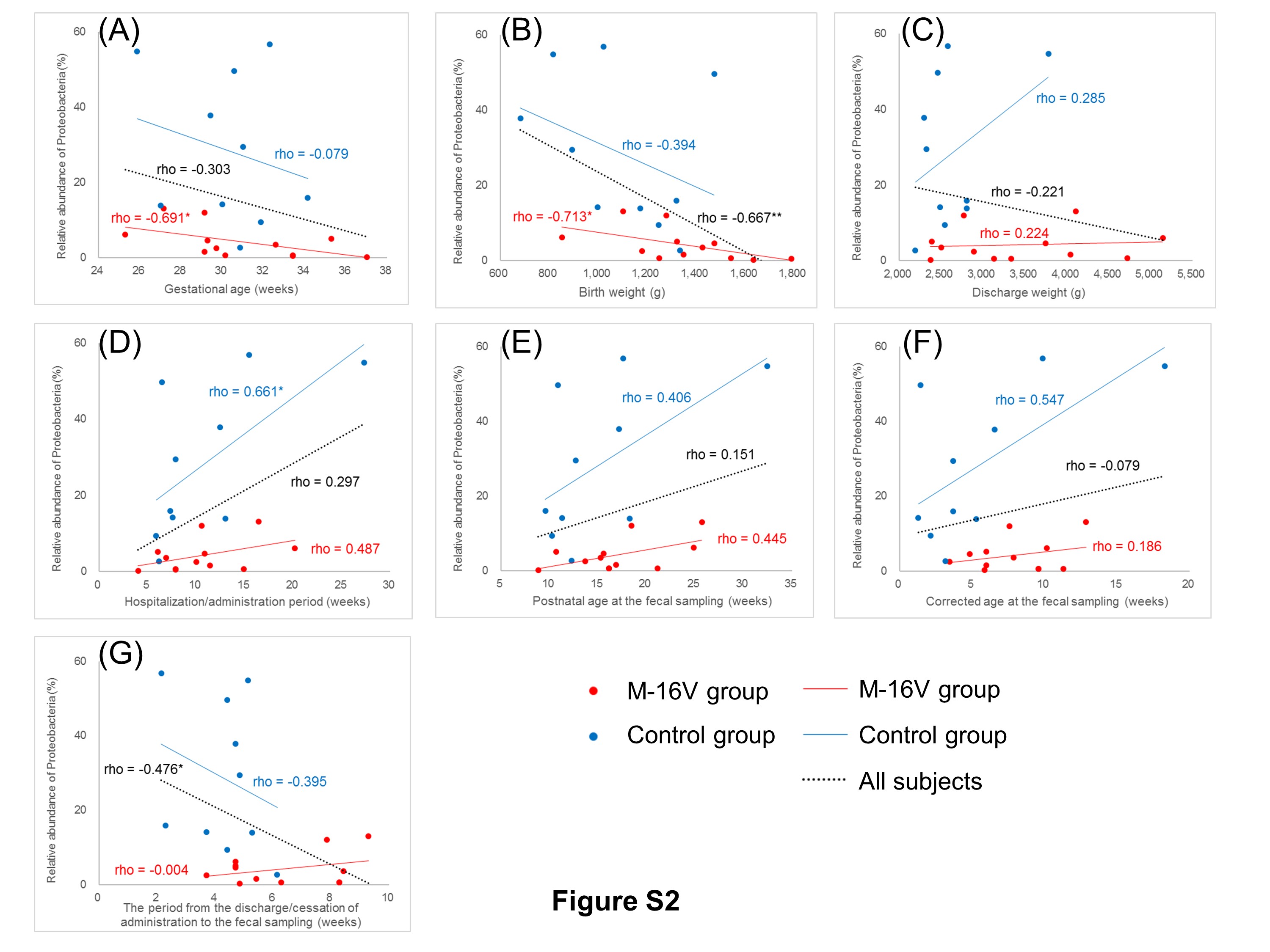

Supplement: Supplementary Figure 2 — Relationship between Proteobacteria and subject’s characteristics. Relationships between the relative abundance of Proteobacteria and the gestational age (A), the birth weight (B), the discharge weight (C), the hospitalization/probiotic administration period (D), the postnatal age at the fecal sampling (E), the corrected age at the fecal sampling (F), or the fecal sampling timing (G) in all subjects (black dashed line), the M-16V group (red circles and line), or the control group (blue circles and line). Associations were assessed by Spearman’s rank correlation test. *P < 0.05; **P < 0.01. [file Image_2.JPEG]

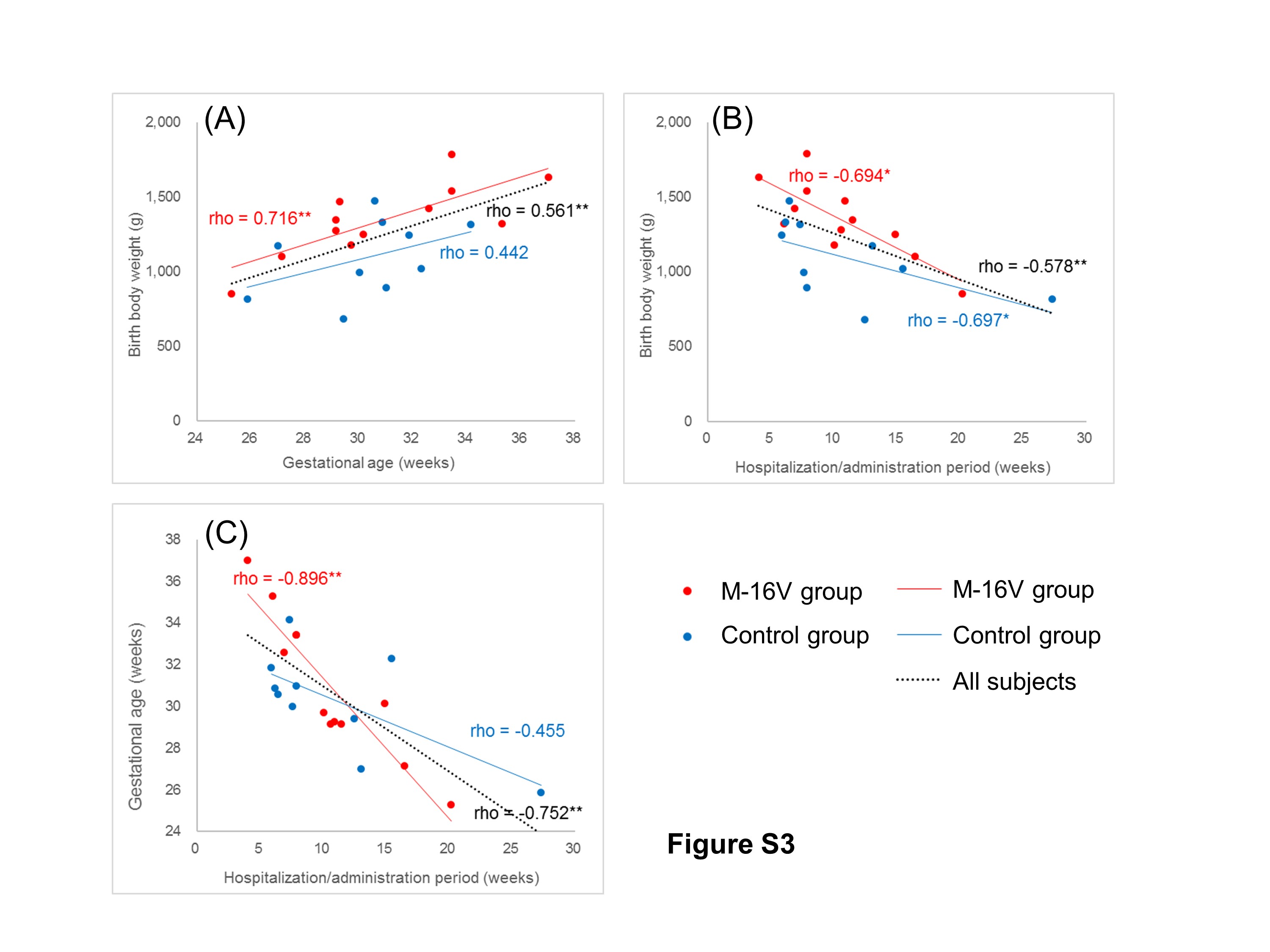

Supplement: Supplementary Figure 3 — Relationship between the birth weight, gestational age, and hospitalization. Relationships between the birth weight and gestational age (A), the birth weight and the hospitalization period (B), and the gestational age and the hospitalization period (C) in all subjects (black dashed line), the M-16V group (red circles and line), or the control group (blue circles and line). Associations were assessed by Spearman’s rank correlation test. *P < 0.05; **P < 0.01. [file Image_3.JPEG]
