# Supplementary material for: Colonization of Supplemented Bifidobacterium breve M-16V in Low Birth Weight Infants and Its Effects on Their Gut Microbiota Weeks Post-administration
Source: Front Microbiol. 2021 Apr 7;12:610080. doi: 10.3389/fmicb.2021.610080 (PMC8058467; doi:10.3389/fmicb.2021.610080)
Supplement: Supplementary Table 1 — Study schedule for each subject. [file Table_1.docx]

**Table S1. Study schedule for each subject**

| Group | Subject No. | Birth | |  | Discharge | | Hospitalization　period (weeks) | Probiotic administration period (weeks) | Fecal sampling | | | |
| --- | --- | --- | --- | --- | --- | --- | --- | --- | --- | --- | --- | --- |
|  |  | GA (weeks) | Weight (g) |  | Date (m/d/y) | Weight (g) |  |  | Date (m/d/y) | The period from the discharge to the fecal sampling (weeks) | Postnatal age (weeks) | Corrected age (weeks) |
| M-16V | 1 | 37.0 | 1636 |  | 5/25/2012 | 2374 | 4.0 | 4.0 | 6/28/2012 | 4.9 | 8.9 | 5.9 |
|  | 2 | 35.3 | 1322 |  | 6/7/2012 | 2392 | 6.0 | 6.0 | 7/10/2012 | 4.7 | 10.7 | 6.0 |
|  | 3 | 32.6 | 1426 |  | 2/24/2013 | 2504 | 6.9 | 6.9 | 4/24/2013 | 8.4 | 15.3 | 7.9 |
|  | 4 | 33.4 | 1790 |  | 11/24/2012 | 3335 | 7.9 | 7.9 | 1/21/2013 | 8.3 | 16.1 | 9.6 |
|  | 5 | 33.4 | 1544 |  | 11/24/2012 | 3130 | 7.9 | 7.9 | 1/21/2013 | 8.3 | 16.1 | 9.6 |
|  | 6 | 29.7 | 1180 |  | 4/26/2017 | 2890 | 10.0 | 10.0 | 5/22/2017 | 3.7 | 13.7 | 3.4 |
|  | 7 | 29.1 | 1280 |  | 1/29/2016 | 2770 | 10.6 | 10.6 | 3/24/2016 | 7.9 | 18.4 | 7.6 |
|  | 8 | 29.3 | 1474 |  | 1/19/2013 | 3744 | 10.9 | 10.9 | 2/21/2013 | 4.7 | 15.6 | 4.9 |
|  | 9 | 29.1 | 1350 |  | 10/20/2012 | 4045 | 11.4 | 11.4 | 11/27/2012 | 5.4 | 16.9 | 6.0 |
|  | 10 | 30.1 | 1250 |  | 12/18/2012 | 4725 | 14.9 | 14.9 | 1/31/2013 | 6.3 | 21.1 | 11.3 |
|  | 11 | 27.1 | 1102 |  | 1/29/2016 | 4110 | 16.4 | 16.4 | 4/3/2016 | 9.3 | 25.7 | 12.9 |
|  | 12 | 25.3 | 852 |  | 2/21/2013 | 5145 | 20.1 | 20.1 | 3/26/2013 | 4.7 | 24.9 | 10.1 |
| Control | 13 | 31.9 | 1247 |  | 11/3/2012 | 2546 | 5.9 | 0.0 | 12/4/2012 | 4.4 | 10.3 | 2.1 |
|  | 14 | 30.9 | 1335 |  | 12/9/2012 | 2196 | 6.1 | 0.0 | 1/21/2013 | 6.1 | 12.3 | 3.1 |
|  | 15 | 30.6 | 1475 |  | 11/3/2012 | 2464 | 6.4 | 0.0 | 12/4/2012 | 4.4 | 10.9 | 1.4 |
|  | 16 | 34.1 | 1319 |  | 10/7/2012 | 2808 | 7.3 | 0.0 | 10/23/2012 | 2.3 | 9.6 | 3.7 |
|  | 17 | 30.0 | 998 |  | 10/5/2012 | 2492 | 7.6 | 0.0 | 10/31/2012 | 3.7 | 11.3 | 1.3 |
|  | 18 | 31.0 | 895 |  | 9/19/2012 | 2330 | 7.9 | 0.0 | 10/23/2012 | 4.9 | 12.7 | 3.7 |
|  | 19 | 29.4 | 683 |  | 12/19/2012 | 2296 | 12.4 | 0.0 | 1/21/2013 | 4.7 | 17.1 | 6.6 |
|  | 20 | 27.0 | 1173 |  | 9/22/2012 | 2806 | 13.0 | 0.0 | 10/29/2012 | 5.3 | 18.3 | 5.3 |
|  | 21 | 32.3 | 1022 |  | 10/7/2012 | 2582 | 15.4 | 0.0 | 10/22/2012 | 2.1 | 17.6 | 9.9 |
|  | 22 | 25.9 | 816 |  | 9/23/2012 | 3780 | 27.3 | 0.0 | 10/29/2012 | 5.1 | 32.4 | 18.3 |
