# Supplementary material for: Colonization of Supplemented Bifidobacterium breve M-16V in Low Birth Weight Infants and Its Effects on Their Gut Microbiota Weeks Post-administration
Source: Front Microbiol. 2021 Apr 7;12:610080. doi: 10.3389/fmicb.2021.610080 (PMC8058467; doi:10.3389/fmicb.2021.610080)
Supplement: Supplementary Table 2 — Quantitative PCR primer sets. [file Table_2.docx]

**Table S2. Quantitative PCR primer sets**

| **Target species/strain** | **Primer** | **Sequence (5' to 3')** | **Reference** |
| --- | --- | --- | --- |
| *Bifidobacterium* spp*.* | g-Bifid-F | CTCCTGGAAACGGGTGG | *****Requena et al., 2002 |
|  | g-Bifid-R | GGTGTTCTTCCCGATATCTACA |  |
| *Bifidobacterium breve* | BiBRE-1 | CCGGATGCTCCATCACAC | Matsuki et al., 1998 |
|  | BiBRE-2 | ACAAAGTGCCTTGCTCCCT |  |
| *Bifidobacterium longum* group | BiLONg-1 | TTCCAGTTGATCGCATGGTC | Matsuki et al., 1998 |
|  | BiLONg-2 | TCSCGCTTGCTCCCCGAT |  |
| *Bifidobacterium longum* subsp. *longum* | BiLON-1 | TTCCAGTTGATCGCATGGTC | **Matsuki et al., 1999 |
|  | BiLON-2 | GGGAAGCCGTATCTCTACGA |  |
| *Bifidobacterium bifidum* | BiBIF-1 | CCACATGATCGCATGTGATTG | Matsuki et al., 1998 |
|  | BiBIF-2 | CCGAAGGCTTGCTCCCAAA |  |
| *Bifidobacterium catenulatum* group | BiCATg-1 | CGGATGCTCCGACTCCT | Matsuki et al., 1998 |
|  | BiCATg-2 | CGAAGGCTTGCTCCCGAT |  |
| *Bifidobacterium adolescentis* group | BiADOg-1a | CTCCAGTTGGATGCATGTC | Matsuki et al., 2004 |
|  | BiADOg-1b | TCCAGTTGACCGCATGGT |  |
|  | BiADOg-2 | CGAAGGCTTGCTCCCAGT |  |
| *Bifidobacterium breve* M-16V | M16Vnew1-F | TAACGGGTCCTATCGTCCAG | This study |
|  | M16Vnew1-R | ATGAGCCGAAAACAGAAGGA |  |

*****Requena, T., Burton, J., Matsuki, T., Munro, K., Simon, M. A., Tanaka, R., et al. (2002). Identification, detection, and enumeration of human bifidobacterium species by PCR targeting the transaldolase gene. *Appl. Environ. Microbiol.* 68, 2420–2427. doi:10.1128/aem.68.5.2420-2427.2002

**Matsuki, T., Watanabe, K., Tanaka, R., Fukuda, M., and Oyaizu, H. (1999). Distribution of bifidobacterial species in human intestinal microflora examined with 16S rRNA-gene-targeted species-specific primers. *Appl. Environ. Microbiol.* 65, 4506–4512. doi: 10.1128/aem.65.10.4506-4512.1999
