# Supplementary material for: Colonization of Supplemented Bifidobacterium breve M-16V in Low Birth Weight Infants and Its Effects on Their Gut Microbiota Weeks Post-administration
Source: Front Microbiol. 2021 Apr 7;12:610080. doi: 10.3389/fmicb.2021.610080 (PMC8058467; doi:10.3389/fmicb.2021.610080)
Supplement: Supplementary Table 3 — Alpha-diversity of the gut microbiota. [file Table_3.docx]

**Table S3. Alpha-diversityof the gut microbiota**

| **Group** | **M-16V group (n=12)** | **Control group (n=10)** | ***P*-value** |
| --- | --- | --- | --- |
| Chao1 index | 30.37 ± 13.18 | 34.65 ± 8.82 | 0.393 |
| Observed OTUs | 30.17 ± 12.79 | 34.40 ± 8.86 | 0.388 |
| Faith’s phylogenetic diversity | 3.46 ± 1.56 | 3.63 ± 0.85 | 0.764 |
| Shannon’s index | 2.20 ± 0.65 | 2.65 ± 0.62 | 0.120 |

Data are expressed as the mean ± SD.Intergroup differences were analyzed using the unpaired Student’s *t*-test. OTUs, operational taxonomic units.
